# Structural and Biochemical Analysis of 3-Dehydroquinate Dehydratase from *Corynebacterium glutamicum*

**DOI:** 10.4014/jmb.2305.05018

**Published:** 2023-08-18

**Authors:** Chan Hwi Lee, Sangwoo Kim, Hogyun Seo, Kyung-Jin Kim

**Affiliations:** 1 School of Life Sciences, BK21 FOUR KNU Creative BioResearch Group, Kyungpook National University, Daegu 41566, Republic of Korea; 2 KNU Institute for Microorganisms, Kyungpook National University, Daegu 41566, Republic of Korea

**Keywords:** 3-Dehydroquinate dehydratase, *Corynebacterium glutamicum* ATCC 13032, shikimate pathway, chorismate biosynthesis

## Abstract

Dehydroquinate dehydratase (DHQD) catalyzes the conversion of 3-dehydroquinic acid (DHQ) into 3-dehydroshikimic acid in the mid stage of the shikimate pathway, which is essential for the biosynthesis of aromatic amino acids and folates. Here, we report two the crystal structures of type II DHQD (*Cg*DHQD) derived from *Corynebacterium glutamicum*, which is a widely used industrial platform organism. We determined the structures for *Cg*DHQD^WT^ with the citrate at a resolution of 1.80Å and *Cg*DHQD^R19A^ with DHQ complexed forms at a resolution of 2.00 Å, respectively. The enzyme forms a homododecamer consisting of four trimers with three interfacial active sites. We identified the DHQ-binding site of *Cg*DHQD and observed an unusual binding mode of citrate inhibitor in the site with a half-opened lid loop. A structural comparison of *Cg*DHQD with a homolog derived from *Streptomyces coelicolor* revealed differences in the terminal regions, lid loop, and active site. Particularly, *Cg*DHQD, including some *Corynebacterium* species, possesses a distinctive residue P105, which is not conserved in other DHQDs at the position near the 5-hydroxyl group of DHQ. Replacements of P105 with isoleucine and valine, conserved in other DHQDs, caused an approximately 70% decrease in the activity, but replacement of S103 with threonine (*Cg*DHQD^S103T^) caused a 10% increase in the activity. Our biochemical studies revealed the importance of key residues and enzyme kinetics for wild type and *Cg*DHQD^S103T^, explaining the effect of the variation. This structural and biochemical study provides valuable information for understanding the reaction efficiency that varies due to structural differences caused by the unique sequences of *Cg*DHQD.

## Introduction

*Corynebacterium glutamicum* is a nonpathogenic, aerobic, gram-positive, and nonsporulating bacterium that is generally recognized as safe microorganisms. *C. glutamicum* was first isolated in 1956 by researchers at Kyowa Hakko [1, 2]. Since the completion of the full genome sequence of *C. glutamicum* ATCC 13032 in 2003 [3, 4], this bacterium has been found to possess several physiological properties suitable for fermentative production. Its most useful properties include the ability to consume high levels of sugar under various conditions [5, 6], tolerance to toxic products [7-9], and consumption of mixed sugars due to innate deficiencies [10-12]. Therefore, *C. glutamicum* has become one of the most widely used microorganisms in industrial biotechnology and microbiology for producing amino acids such as L-glutamate and L-lysine [13-17]. Furthermore, it is valuable for producing aromatic compounds in the bioindustry [18-21]. In a recent research, shikimate was overproduced in engineered *C. glutamicum* [22].

The shikimate pathway is present in bacteria, fungi, archaea, algae, some protozoans, and plants. It is a seven-step metabolic process that converts phosphoenolpyruvate and D-erythrose-4-phosphate into chorismite, which is the precursor of aromatic amino acids such as L-phenylalanine, L-tyrosine, and L-tryptophan, as well as other aromatic metabolites such as quinones, lignin, and flavonoids [23-25]. Hence, this pathway is an attractive target for food additives, hazardous chemicals, and biopharmaceuticals [23, 26-28]. However, it has also been considered a valuable resource for the development of antibacterial agents due to its absence in humans. Today, metabolic engineering strategies for the shikimate pathway are continually being investigated [29, 30]. Effective methods for fermentative shikimate production have already been developed using engineered microbial strains to achieve large-scale production [31-33].

**Fig. 1:**
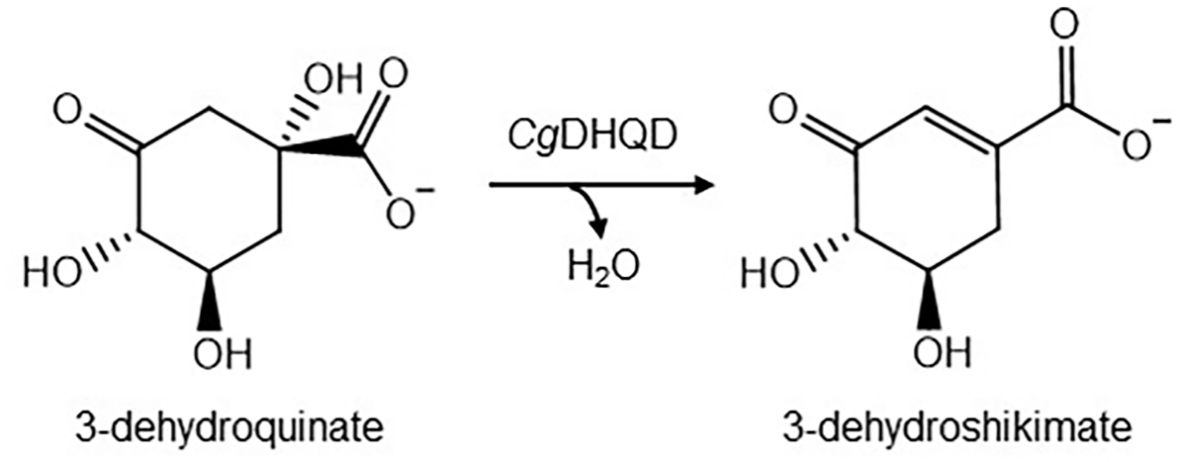
Reaction of *Cg*DHQD. Diagram depicting the catalytic reaction scheme of DHQD.

3-Dehydroquinate dehydratase (DHQD, DHQase, E.C. 4.2.1.10)) is involved in the third step in the shikimate pathway, catalyzing the dehydration of 3-dehydroquinic acid (DHQ) to 3-dehydroshikimic acid (DHS) (Fig. 1A). This enzyme is classified into two distinct classes [23, 34, 35]. Type I DHQD, corresponding to *aroD*, catalyzes *syn*-dehydration through a covalent imine intermediate [33, 36]. Type II DHQD, encoded by *aroQ*, catalyzes *anti*-dehydration by forming a Schiff base with the conserved Lys residue through an enolate intermediate [37]. The two types of DHQD differ in terms of structural characterization. Type I enzymes, which have (α/β)8 fold, exist as homodimers [38, 39]. Type II enzymes exist as homododecamers containing a flavodoxin fold [35, 40].

In this study, we conducted structural and biochemical investigations on *Cg*DHQD and present the findings on two crystal structures of type II DHQD, including the citrate inhibitor and DHQ complexed form, with high-resolution data (1.8 and 2.0 Å). On the basis of these structures, we identified differences from other DHQD sequences. In particular, we found that the unique proline residue at location 105, found only in some of the *C. glutamicum* family, plays a vital role in protein activity. We also examined the effects of key residue variations in *Cg*DHQD and the kinetic behavior of the enzyme and its variant. This study will provide structural and biochemical implications of the key component of the microbial shikimate pathway.

## Materials and Methods

### Protein Expression and Purification

The *Cg*DHQD genomic code was amplified through polymerase chain reaction (PCR) using primers: forward 5’-GCGCCATATGCCTGGAAAAATTCTCCTCCTCAAC, reverse 3’-GCGCCTCGAGCTTTTTGAGATTTG CCAGGATATC, respectively. The amplified products were digested by NdeI and XhoI restriction enzymes. The DNA fragment was cloned into a pET30a expression vector (Merck Millipore, USA), which contains a 6x-His tag at the C-terminal. pET30a:*Cg*DHQD was transformed into *E. coli* BL21 (DE3)^T1R^ strain. The cells were cultured in a fresh LB medium containing 50 mg/l kanamycin at 310 K. After the OD_600_ of culture media reached 0.6, 0.5 mM isopropyl-1-thio-beta-D-galactopyranoside (IPTG) was added to the cells to induce protein expression, followed by further incubation at 291 K for 20 hours. After harvest, the cell pellet was resuspended in lysis buffer (40 mM Tris-HCl, pH 8.0). Site-directed mutations were performed using a QuikChange kit (Agilent, USA), and sequencing was performed to confirm correct incorporation of the mutations. The variants were purified in the same manner as wild type *Cg*DHQD.

The cell debris was disrupted by ultrasonication and removed by centrifugation at 13,000 ×*g* for 30 min, and lysate was bound to Ni-NTA agarose (Qiagen, Germany). After washing with the lysis buffer containing 20 mM imidazole, the bound proteins were eluted with a lysis buffer containing 300 mM imidazole. The *Cg*DHQD protein was purified by ion-exchange chromatography (HiTrap Q FF, GE Healthcare, USA) and size exclusion chromatography (HiPrep 26/60 Sephacryl S-300 HR column, GE Healthcare) for further purification. The purified protein was concentrated to 40 mg/ml in 40 mM Tris-HCl, pH 8.0.

### Crystallization

The purified protein was initially crystallized using sparse-matrix screens, including Index, PEG ion I and II (Hampton Research), Structure screen I and II (Molecular Dimensions) and Wizard Classic I and II (Rigaku Reagents). Crystals were grown using the sitting drop vapor diffusion method at 293K. Each experimental drop consisted of mixing 1.0 μl of purified protein solution (40 mg/ml, 40 mM Tris-HCl, pH 8.0) with 1.0 μl reservoir solution and equilibrating it against 50 μl of reservoir solution. Before collecting X-ray diffraction data, the best crystal was transferred to cryoprotectant crystallization reservoir solution containing 20% PEG 3350 and 0.2 M ammonium citrate tribasic, pH 7.0 and 30% glycerol. The crystals of *Cg*DHQD complexed with citrate were obtained from the condition of 20% PEG 3350 and 0.2 M ammonium citrate tribasic, pH 7.0. The crystals of *Cg*DHQD^R19A^ were grown under the conditions of 0.8 M lithium sulfate, 0.4 M ammonium sulfate, and 0.1 M sodium citrate, pH 5.5 and soaked in a solution of the condition containing 100mM DHQ. Before collecting X-ray diffraction data, the best crystal was transferred to cryoprotectant crystallization reservoir solution containing of 0.8 M lithium sulfate, 0.4 M ammonium sulfate, and 0.1 M sodium citrate, pH 5.5 and 30% glycerol.

### Data Collection and Structure Determination

Data were collected at 100K at the 7A beamline of the Pohang Accelerator Laboratory (PAL, Repubilc of Korea), using a QUANTUM 270 CCD detector (USA) [41]. All data were then indexed, integrated and scaled together using the HKL 2000 software package [42]. Crystals of the *Cg*DHQD citrate complexed form belonged to the trigonal space group R3, collected to a resolution of 1.80 Å, with unit cell parameters of *a = b =* 81.5, *c =* 221.8. Assuming four molecules of citrate complexed form per asymmetric unit, the crystal volume per unit of protein mass was 2.03 Å3 Da^-1^, which corresponded to a solvent content of approximately 39.03% [43]. Crystals of *Cg*DHQD^R19A^ complex belonged to the orthorhombic space group *C*222_1_, collected to a resolution of 2.0 Å, with unit cell parameters of *a =* 150.6, *b =* 165.3, *c =* 106.5. Assuming six molecules of the complex with DHQ per asymmetric unit, the crystal volume per unit of protein mass was 3.19 Å3 Da^-1^, which corresponded to a solvent content of approximately 61.08% [43]. The *Cg*DHQD citrate complexed form structure was determined by molecular replacement with MOLREP, using the monomer structure of type II DHQD from *Streptomyces coelicolor* (PDB code 1V1J, 58.27% sequence identity) as a template model [44, 45]. Further model building was performed manually using the program WinCoot, and refinement was performed with REFMAC5 in CCP4 Package [46-48]. The data statistics were summarized in Table 1. The refined model was deposited in the Research Collaboratory for Structural Bioinformation (RCSB) Protein Data Bank (PDB) codes of 8IDR and 8IDU.

The tertiary structures of *Cg*DHQD^S103T^, *Cg*DHQD^P105V^, and *Cg*DHQD^P105I^ mutants are predicted by using Colabfold v1.5.1 : Alphafold2 using MMseqs2 [49]. Among the five models, the structure closest to the closed form was selected. In order to visualize the molecular structures of wild type and mutants, we utilized the Pymol program, which enabled us to generate informative and visually figures that highlighted structural features and interactions [50].

### Size-Exclusion Chromatography

In order to verify the oligomeric status of *Cg*DHQD, analytical size-exclusion chromatography was conducted using a Superdex 200 Increase 10/300 GL column (Cytiva). The column was equilibrated with 150 mM NaCl and 40 mM Tris–HCl, at pH 8.0. A protein sample utilized for the analysis was 1ml in volume and had a concentration of 1 mg/ml. To determine the molecular weight of the eluted *Cg*DHQD sample from the column, a calibration curve was generated using standard samples of aldolase (158 kDa), ovalbumin (44 kDa), ribonuclease A (13.7 kDa), and aprotinin (6.5 kDa). All purification experiments were conducted at temperature of 277K to ensure consistency in the experimental conditions.

### Phylogenetic Tree and Conservation Logo Analysis

To investigate for comparison with other specifications, we used the Basic Local Alignment Search Tool (BLAST) search in National Center for Biotechnology Information (NCBI) server [51]. We applied the Position-Specific iterated BLAST (PSI-BLAST) algorithm to select 5000 sequences for alignment [52], and performed multiple sequence alignment using the *Clustal Omega* program [53].

The evolutionary relationships of 501 amino acid sequences, including DHQD from *Streptomyces coelicolor* (*Sc*DHQD) [54], were analyzed using MEGA11 software [55]. The tree with the highest logo likelihood value (-18374.23) was selected, and distances were estimated using a LG model. A discrete Gamma distribution was used to model evolutionary rate differences among sites (G parameter: 0.75). The rate variation model allowed for some sites to be evolutionarily invariable. Branch lengths were measured in the number of substitutions per site, and the tree was drawn to scale. Positions with less than 95% site coverage were eliminated, allowing for fewer than 5% alignment gap, missing data, and ambiguous bases at any position.

In addition, we utilized the PSI-BLAST algorithm to select 750 multispecies homologs, including sequence of *Cg*DHQD, from a pool of the 5000 homologs *Cg*DHQD. This selection was made to generate a sequence logo specifically for the active site. The sequence logo displayed the representative residues of the active site of DHQDs. The Weblogo 3 software was used to create this logo [56].

### Activity Measurement

The experiment aimed to measure the activity of *Cg*DHQD wild type and its variants by monitoring the increased absorbance value (ε = 1.2 × 10^4^ M^-1^cm^-1^) of 3-dehydroshikimic acid (DHS) in 234 nm, using an ultraviolet spectrophotometer [57]. A reagent called ‘3-dehydroquinic acid potassium salt (CAS No. 494211-79-9)’ was used for the assay experiment. The reaction mixture was comprised of 50 mM Tris-HCl, pH 8.0, and substrate, with the concentration ranging from 50 to 2,000 μM. Following a one-minute incubation period at room temperature, the enzyme was added to initiate the reaction, and the assay was conducted with an enzyme concentration of 20 nM.

### Kinetic Parameter Determination of *Cg*DHQD^WT^ and *Cg*DHQD^S103T^

The statistical analysis of V_max_, K_M_ and k_cat_ values were carried out using Michaelis Menten models with OriginPro 2023 software (Originlab Corporation, USA). To compare the relative activity of the mutants with that of wild type, mutant proteins were purified using same procedure as the wild type *Cg*DHQD. The activity of other variants was measured using 20 μM enzymes and 2,000 μM substrate. All assay experiments were conducted in triplicate at room temperature, with a reaction mixture of 0.5 ml to obtain idealized data.

## Results and Discussion

### Overall Structure of *Cg*DHQD

To define the structural features and molecular functions of *Cg*DHQD, we determined its crystal structures of citrate inhibitor and DHQ complexed forms at 1.80 and 2.00 Å resolution, respectively. The refined structures were in good agreement with crystallographic statistics for bond angles, lengths, and other geometric parameters (Table 1). The asymmetric unit of the citrate- and DHQ-bound structures contained four and six molecules, respectively, highly homologous with alpha carbon, with root mean square deviation values within 0.2.

**Fig. 2:**
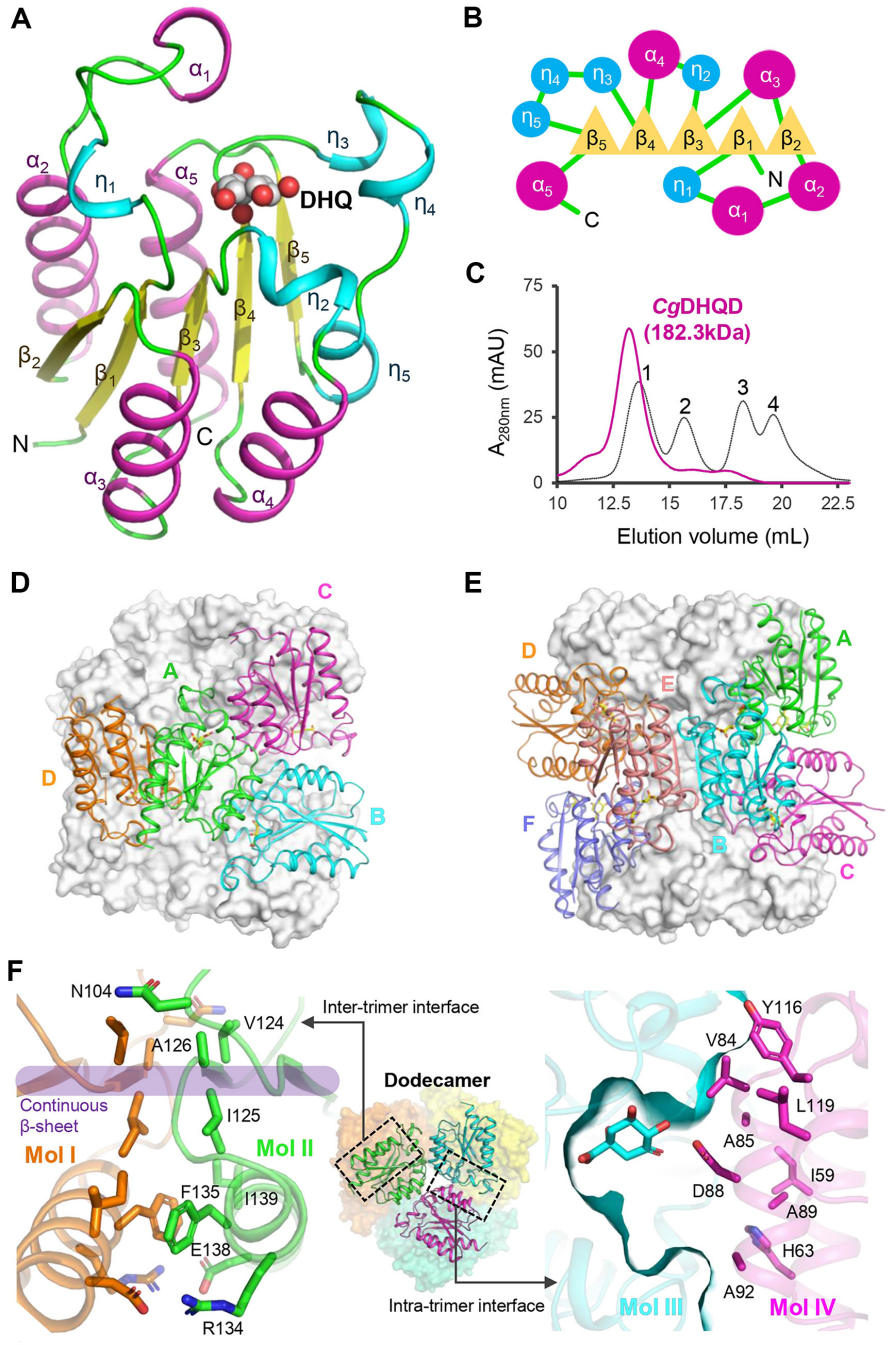
Oligomeric status of *Cg*DHQD. (**A**) Monomer structure of *Cg*DHQD. The monomer is shown as a cartoon model, with the DHQ molecule presented by spheres. The α-helices, 3_10_-helices, and β-strands are colored in magenta, cyan, and yellow, respectively, and labeled. (**B**) Schematic of *Cg*DHQD. The α-helices, 3_10_-helices, and β-strands are depicted as circles, triangles, and lines, respectively. The color scheme is the same as in Fig. 1A. (**C**) Analysis of *Cg*DHQD using size exclusion chromatography. The magenta color highlights *Cg*DHQD. The standard samples of aldolase (158kDa), ovalbumin (44kDa), ribonuclease A (13.7 kDa), and aprotinin (6.5 kDa) are labeled 1, 2, 3, and 4, respectively. (**D**) Overall structure of citrate complexed form of *Cg*DHQD. (**E**) Overall structure of DHQ complexed form of *Cg*DHQD^R19A^. (**F**) Interface of *Cg*DHQD. The structure of *Cg*DHQD is shown as a dodecameric cartoon model. The purple color highlights continuous β-strands in the intratrimer interface. The residues involved in the intertrimer and intratrimer interactions are shown as stick models and labeled.

Our analysis revealed that the monomer is composed of repetitive α/β domains, forming a flavodoxin fold with central parallel β sheets arranged in a specific order (Fig. 2C and 2D). The active site of *Cg*DHQD is located at the end of the central β sheets and surrounded by loops containing 3_10_-helices (Fig. 2C). The α1 is a lid loop region that adopts a conformational change of open and closed forms [34]. To analyze the oligomeric status, *Cg*DHQD was eluted at a molecular mass of 182.3 kDa through a size exclusion chromatography experiment, which indicated that *Cg*DHQD forms a dodecamer in solution as other known DHQDs [34] (Fig. 2E).

The dodecameric structure of *Cg*DHQD is composed of four homotrimers with a tetrahedral symmetry (Fig. 2F). The intertrimer interface forms a solvent accessible area of 723.9 Å2 of −4.8 kcal/mol solvation free energy gain (Δ^i^G) computed by PISA [58]. The strong network includes continuous hydrogen bonds of the β sheet, salt bridges between R134 and E138, and van der Waals interactions of V124, I125, A126, F135, and I139 (Fig. 2F). In contrast, the intratrimer interface is primarily facilitated by hydrophobic interactions with a solvent accessible area of 686.5 Å2 and a Δ^i^G value of −4.6 kcal/mol (Fig. 2F). The interface contains D88 that is involved in the formation of the active site (Fig. 2F).

### Active Site of *Cg*DHQD

**Fig. 3:**
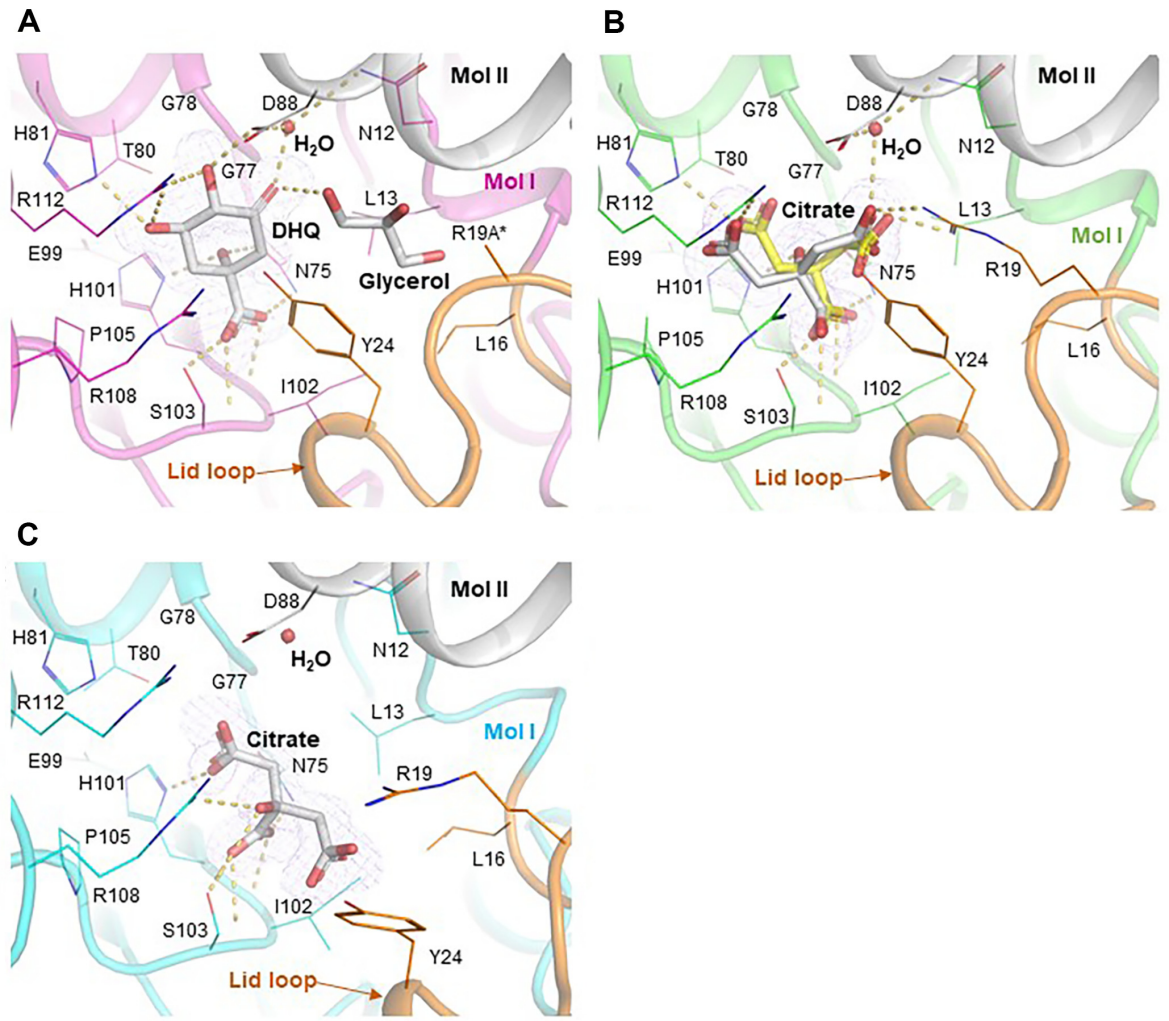
Active site of *Cg*DHQD. The active site of *Cg*DHQD. The *F*_o_−*F*_c_ electron density map is displayed as a purple mesh contoured at 3.5σ. (**A**) The complexed form with the substrate. The catalytic pocket of *Cg*DHQD with DHQ is shown as a stick model colored in magenta. DHQ and glycerol are colored in white. (**B**) The general complex form with citrate. The catalytic pocket of *Cg*DHQD with the general citrate complexed form is shown by colored green. The yellow citrate molecule is prepared from *Helicobacter pylori* (PDB code: 2C4V) by alpha carbon alignment with *Cg*DHQD. (**C**) The uncommon complexed form with citrate. The uncommon catalytic pocket of the *Cg*DHQD-complexed form is shown and colored in cyan. The lid loop and neighboring chains of the figures are colored in orange and white, respectively. The yellow dotted line indicates the polar interaction contributing to the binding chemicals.

The DHQ-bound structure of *Cg*DHQD explained the substrate-binding mode of the enzyme. The substrate is stabilized by hydrogen bonds with a water molecule, side chains of N75, H81, D88, H101, S103, and R112, and main chains of I102 and S103 (Fig. 3A). Moreover, the neighboring residues L13, G77, G78, T80, and P105 organize the substrate-surrounding environment of the active site (Fig. 3A). A glycerol molecule exists in this structure to occupy the space of R19A variation that eliminates the enzyme activity for crystallographic benefit. There are six residues that directly participate in enzyme catalysis, including R19, Y24, N12, E99, H101, and R108 [59]. The Y24 positions toward the 2-carbon of DHQ to function as a general base catalyst with R19 and R108 activators (Fig. 3A). The position of the water molecule is known to be crucial, and it mediates the initial electron transfer from Y24 to N12 [59]. H101 and E99 give a proton to the leaving 1-hydroxyl group of DHQ [59].

Studies on DHQD inhibitors have provided valuable information for the development of herbicides and antimicrobial agents [32, 60-62]. The citrate ion exerts a weak inhibitory effect on DHQD, which is enzyme-dependent [61]. The *Cg*DHQD structure in complex with the citrate ion exhibits two different binding modes of the molecule. The citrate ions bound in two *Cg*DHQD chains in the asymmetric unit are stabilized by the side chains of R19, Y24, N75, H81, H101, S103, and R112 and the main chains of S103 and I102 (Fig. 3B). The citrate-binding mode is similar in that the stabilization of 3-carboxyl and 3-hydroxyl groups resembles that of 1-carboxyl and 1-hydroxyl groups of DHQ (Fig. 3B), as observed in the structure of *Helicobacter pylori* DHQD [61]. However, the other binding mode of citrate in the other chains in the asymmetric unit is uncommon, wherein the molecule is accommodated in the active site with a half-opened lid loop (Fig. 3C). In this mode, the 3-hydroxyl group of the citrate molecule is rotated by almost 180° compared to that in the general binding mode, and the molecule does not interact with the residues R19, Y24, H81, and R112, which are essential in the general binding mode. This unique binding mode illustrates the possibility of various interactions for inhibitor binding that do not mimic perfectly fitted substrate binding.

### Sequence and Structural Features of *Cg*DHQD

**Fig. 4:**
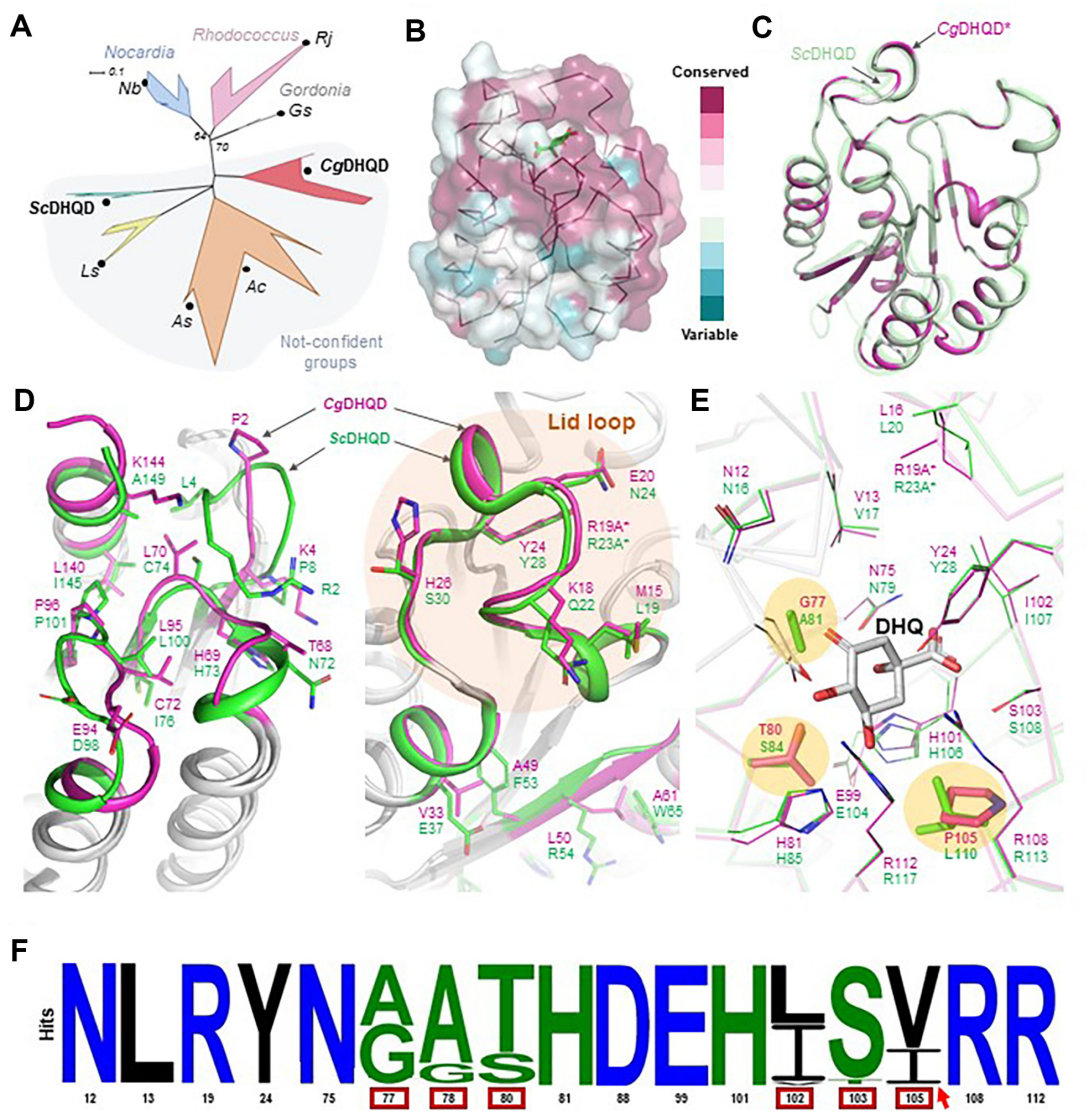
Structural and consensus sequence comparison of *Cg*DHQD. (**A**) The phylogenetic tree for grouping DHQD. Representative DHQDs are marked by black circles and labeled as follows: *Cg*DHQD, WP_003860435.1; *Sc*DHQD, WP_003976858.1; *Ls*, WP_244685684; *As*, WP_219885677; *Ac*, WP_043846238; *Gs*, WP_205330277; *Nb*, WP_167465882; *Rj*, WP_073360406. Significant bootstrap values of 100 replicates are shown on nodes. (**B**) Surface conservation of *Cg*DHQD. The surface conservation of *Cg*DHQD visualized by showing the structure with ribbon and surface models with gradient colors of burgundy and teal. (**C**-**E**) Structural difference between *Cg*DHQD and *Sc*DHQD. (**C**) *Cg*DHQD and *Sc*DHQD are depicted as cartoon models with gray and green colors, respectively. The nonidentical residue points are colored in magenta. (**D**) *Cg*DHQD and *Sc*DHQD are depicted as gray cartoon models. Different regions of *Cg*DHQD and *Sc*DHQD are distinguished with magenta and green colors, respectively. Residues at the region are shown with a stick model and labeled. (**E**) *Cg*DHQD and *Sc*DHQD are depicted as ribbon models colored in magenta and green. The active site residues are shown with a line model and labeled. Three residual differences are distinguished with the stick model and highlighted with orange circles. (**F**) Sequence logos of 750 DHQDs. The numbers below indicate the residue number of *Cg*DHQD at the corresponding point. Variable regions, including G77, G78, T80, I102, S103, and P105, are indicated by red boxes, and P105 is highlighted with a red arrow. Logo plots of the consensus sequence are constructed using Weblogo version 2.8.2. [56].

A phylogenetic tree analysis of *Cg*DHQD homologs revealed some distinguished groups among Actinobacteria, and a structure of *Streptomyces coelicolor* DHQD (*Sc*DHQD) was included in one of the groups (Fig. 4A). Residue conservation analysis of eight representatives of the groups, such as DHQDs from *Leucobacter* sp. H25R-14, *Arthrobacter* sp. PAMC25284, *A. crystallopoietes*, *Gordonia* sp. PDNC005, *Nocardia brasiliensis*, and *Rhodococcus jostii*, using Consurf [63] demonstrated high conservation at the active site region among the DHQDs (Fig. 4B). Superposition between the *Cg*DHQD and *Sc*DHQD structures displayed a highly similar flavodoxin fold, but substantial residual differences were found throughout the structure (Fig. 4C). The residual differences between *Cg*DHQD and *Sc*DHQD structures caused several changes in backbone conformations at regions of N- and C-termini, such as shorter N-terminus, K144 at the C-terminus, an E94–P96 loop, and a T68–C72 loop in *Cg*DHQD (Fig. 4D). At the lid loop region, *Cg*DHQD has M15, K18, E20, and H26 residues at the positions of L19, Q22, N24, and S30 in *Sc*DHQD, respectively, and the complementary networks of V33:A49 and L50:A61 are different from the corresponding points of *Sc*DHQD (Fig. 4D). Although DHQDs have high conservation at residues constituting the active site, we observed replacements of Gly77, Thr80, and Pro105 with alanine, serine, and leucine, respectively, near the DHQ position in *Sc*DHQD (Fig. 4E). These differences affect enzyme activity by altering the substrate-binding site. Further conservation analysis of the 17 active site residues among 750 DHQD sequences revealed some variations at the G77, G78, T80, I102, S103, and P105 positions in *Cg*DHQD (Fig. 4F). P105 was found to be extremely rare in other DHQDs, except *Corynebacterium* DHQDs (Fig. 4F).

### Effects of Conserved Key Residue Variations in *Cg*DHQD

**Fig. 5:**
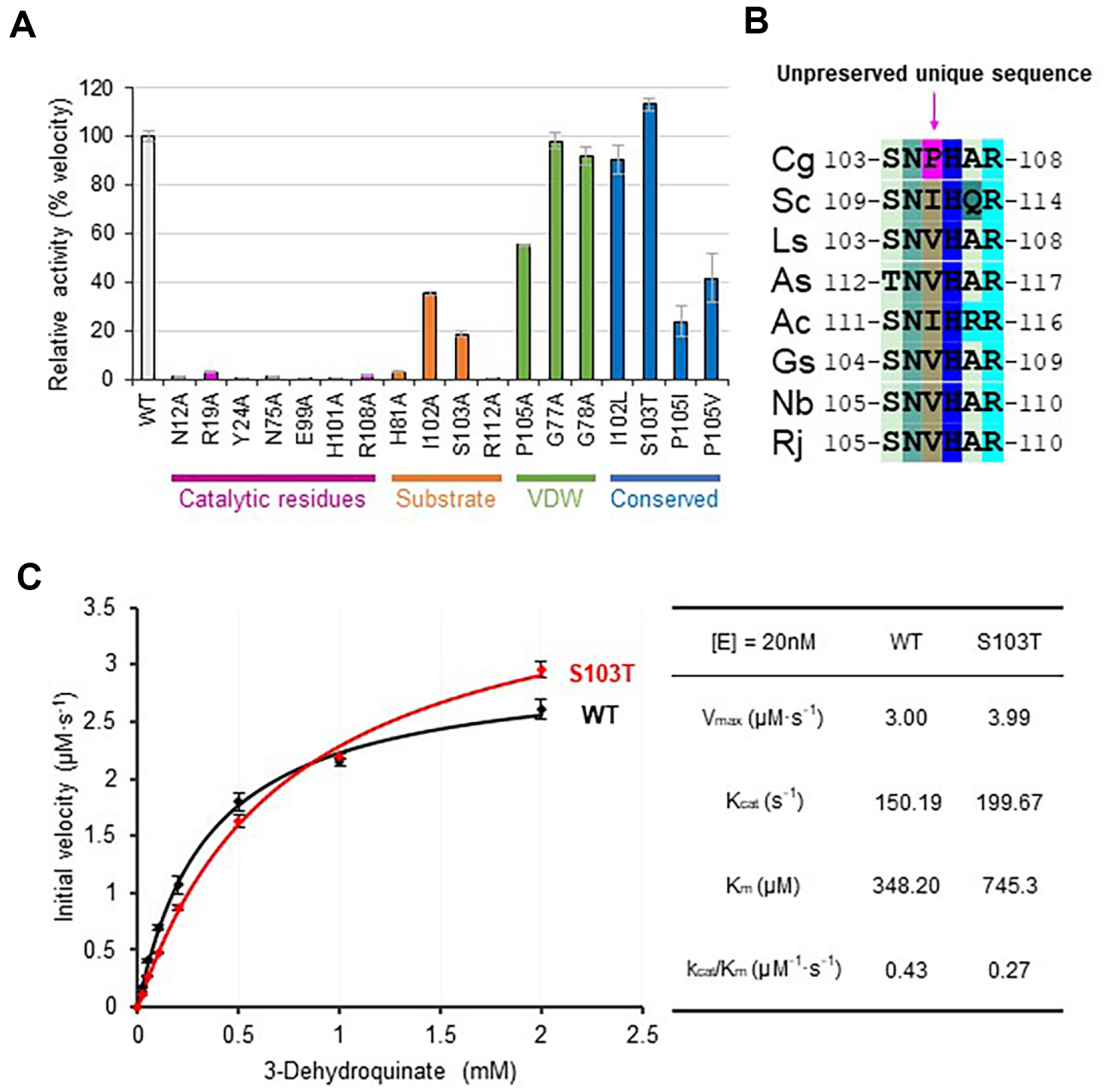
Conservation and biochemical analyses of *Cg*DHQD. (**A**) The activities of wild type and mutants from *Cg*DHQD. To ensure reproducibility of the results, the assays were performed in triplicates. (**B**) P105 region sequence alignment of the eight representative DHQDs. (**C**) Michaelis–Menten plots of *Cg*DHQD^WT^ wild type and *Cg*DHQD S103T. The determined kinetic parameters are summarized in the right table.

We then examined the influence of key residues in *Cg*DHQD on its activity by alanine replacements. Mutations of the catalytic residues, such as N12, R19, Y24, N75, E99, H101, and R108, with alanine resulted in almost complete loss of enzyme activity, conforming that they are crucial for activity in *Cg*DHQD (Fig. 5A). Replacements of residues H81 and R112, which play a significant role in substrate binding, with alanine also resulted in no detectable activities (Fig. 5A). However, I102A and S103A resulted in activities of 35% and 19%, respectively (Fig. 5A). Alanine replacement of the unique P105 residue decreased the activity of *Cg*DHQD by 60%, whereas those at G77 and G78 positions exhibited no meaningful change (Fig. 5A). We next replaced I102, S103, P105, and P105 with other major residues observed in the 750 DHQD sequences and evaluated their relative activities. I102L exhibited a similar level of activity compared to that in the wild type; however, S103T exhibited an increase in activity by >10% (Fig. 5A). Meanwhile, both P105I and P105V showed a significant decrease in activity by 76%and 58%, respectively (Fig. 5A). *Cg*DHQD is remarkable for the exclusive presence of proline residue at this position, a remarkable feature of *Corynebacterium* DHQDs, among the eight representatives in the phylogenetic tree (Fig. 5B). The replacement of the proline residue causes a dramatic decrease in activity compared with the replacements of other nonconserved sites. The presence of the residue at this position is essential for *Cg*DHQD group of enzymes to form optimal active sites.

We also evaluated the kinetic parameters of the *Cg*DHQD wild type and S103T variant (*Cg*DHQD^S103T^), which displayed an enhanced activity of 10%, using the Michaelis–Menten plot (Fig. 5C). The *V*_max_, *K*_m_, and *K*_cat_ values of the wild type were 3.00 μM/s, 348.20 uM, and 150.19 1/s, respectively (Fig. 5C). *Cg*DHQD^S103T^ exhibited *V*_max_, *K*_m_, and *K*_cat_ values of 3.99 μM/s, 745.3 uM, and 199.67 1/s, respectively (Fig. 5C). The 10% increase in *Cg*DHQD^S103T^ activity was due to its increased turnover rate, but it appears to compensate for the decreased substrate affinity.

### Structural Comparison of Key Residue Variations in *Cg*DHQD

We utilized Colabfold software V1.5.1 program to compare the wild type and mutants of *Cg*DHQD. The superimposition of *Cg*DHQD^WT^ with the mutants revealed root-mean-square deviation (RMSD) values of 0.337 Å and 0.323 Å for P105V and P105I. Within a unique proline site, we observed structural variations in the helix-loop-helix (HLH) region (N104-H113). Specifically, the RMSD values for backbone atoms in the helix-loop-helix region of *Cg*DHQD^P105V^ and *Cg*DHQD^P105I^ were 0.233 Å and 0.270 Å, respectively.

**Fig. 6:**
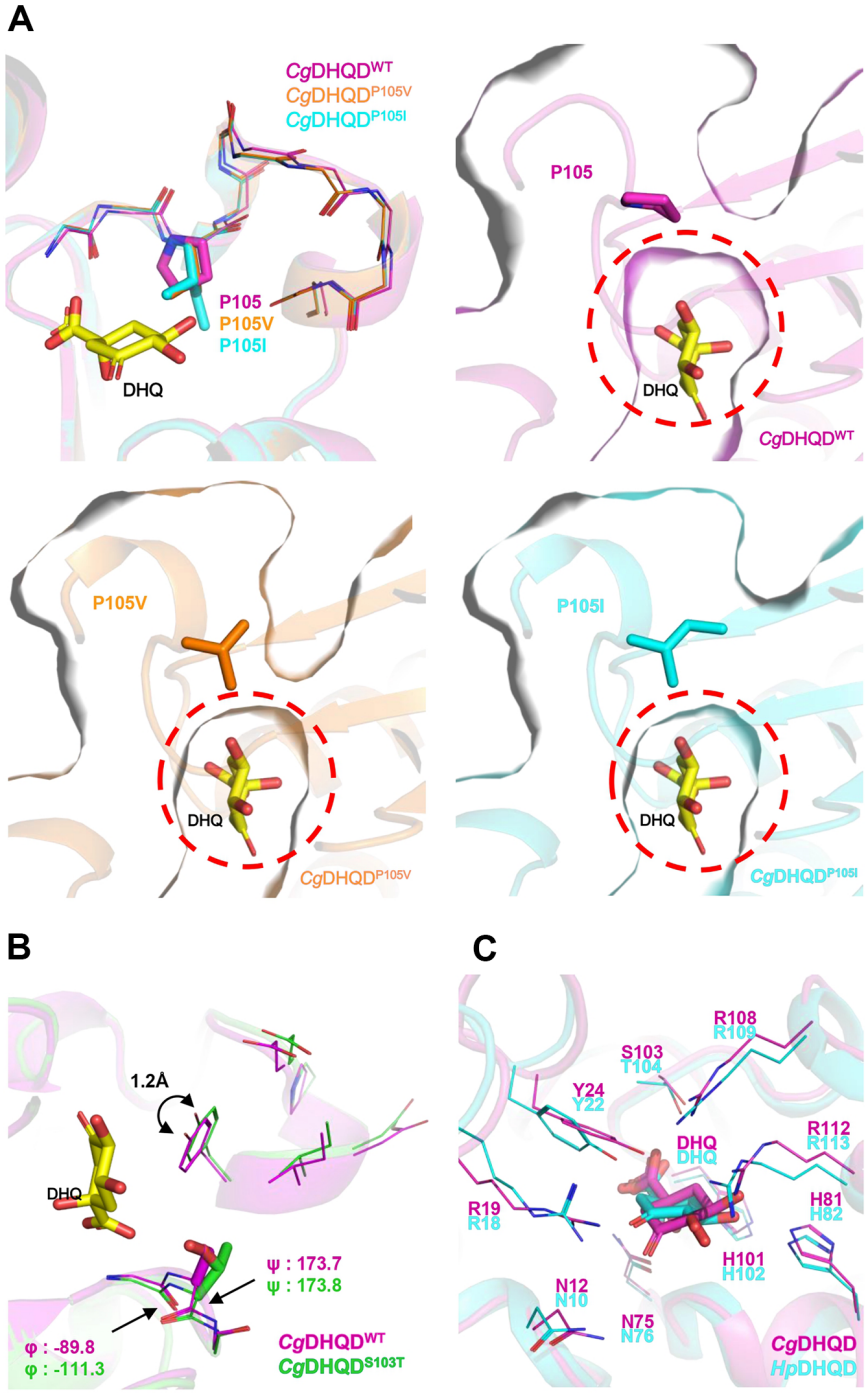
Structural comparison among *Cg*DHQD^WT^, *Cg*DHQD^mutants^ and *Hp*DHQD. (**A**) The main chain and side chain of wild type, P105I and P105V are represented by magenta, orange and cyan colored cartoon and line models, respectively. The DHQ molecules bound to wild type and mutants of *Cg*DHQD are shown in yellow. A dot-line circle is included for comparison with the active site pocket of *Cg*DHQD^WT^, *Cg*DHQD^P105V^, and *Cg*DHQD^P105I^. (**B**) The side chain of *Cg*DHQD^WT^ and *Cg*DHQD^S103T^ are colored in magenta and green, respectively. A DHQ molecule colored in yellow. (**C**) The active site of *Cg*DHQD and *Hp*DHQD (PDB: 1J2Y) are represented with DHQ in magenta and cyan colored cartoon model.

Substituting proline with valine or isoleucine resulted in structural changes in the main chain of the HLH domain, disrupting its binding structure and leading to decreased activity. The wild type with P105 exhibited a larger active site pocket compared to the P105I and P105V mutants (Fig. 6A), which displayed smaller pockets. The wild type demonstrated a more suitable and favorable combination of substrate and protein. However, the P105I and P105V mutations were predicted to have reduced binding affinity for enzymes, as indicated by the smaller binding site pocket (Fig. 6A).

Moreover, we observed that the S103T mutant had a larger active site pocket than the wilt type (Fig. 6B). The substitution of serine with threonine caused changes in the dihedral angle of the main chain residue. In the wild type, the phi angle was -89.8 and the psi angle was 173.7, while the S103T mutant displayed a phi angle of -111.3 and a psi angle of 173.8. Furthermore, the side chain variation from serine to threonine resulted in structural modifications in the lid loop region, pushing the mutant approximately 1.2 Å beyond the position of the wild type. We suspect that the enlarged substrate binding site by the S103T mutation caused decreased affinity for the substrate, resulting in the increased *K*_m_ value.

In comparing *Cg*DQHD^WT^ and *Hp*DHQD^WT^, we observed differences in the induced fit. While most of the residues in the active site were conserved, structural variances in the HLH region influenced the binding mode of the substrate DHQ (Fig. 6C).

In summary, our comprehensive structural and biochemical investigations of *Cg*DHQD derived from the industrial strain of *Corynebacterium* have revealed inhibitor-binding and substrate-binding modes, as well as structural features of the protein. Moreover, we have elucidated the significance of the key residues and conducted a thorough evaluation of the kinetic behavior of both the wild type and *Cg*DHQD^S103T^ variant. These findings offer valuable biochemical insights into this specific protein group, opening possibilities for manipulating microbial biosynthesis of aromatic compounds through the application of inhibitor and protein engineering techniques.

## Figures and Tables

**Table 1:** Data collection, phasing, and refinement statistics.

	*Cg*DHQD	
	Citrate complexed	DHQ complexed
PDB code	8IDR	8IDU
Data collection		
Space group	*R*3	*C*222_1_
Cell dimensions		
*a, b, c* (Å)	81.5, 81.5, 221.8	150.6, 165.3, 106.5
α, β, γ (°)	90.0, 90.0, 120.0	90.0, 90.0, 90.0
Resolution (Å)	50.00-1.80 (1.83-1.80)	50.00-2.03 (2.03-2.00)
*R*_sym_ or *R*_merge_	31.1 (6.5)	33.8 (13.0)
*I* /σ*I*	45.3 (8.2)	30.5 (8.9)
Completeness (%)	99.7 (100.0)	98.6 (97.8)
Redundancy	5.4 (5.3)	9.0 (8.2)
Refinement		
Resolution (Å)	26.51-1.80	30.92-2.00
No. reflections	47482	83591
*R*_work_ / *R*_free_	15.8 / 20.3	14.2 / 16.9
No. atoms	5026	7409
Protein	4372	6389
3-dehydroquinate	-	78
Citrate anion	52	-
Tetraethylene glycol	13	-
Glycerol	-	54
Sulfate ion	-	45
Water	589	843
*B*-factors	26.5	17.8
Protein	25.1	15.5
3-dehydroquinate	-	15.6
Citrate anion	32.5	-
Tetraethylene glycol	45.5	-
Glycerol	-	39.4
Sulfate ion	-	69.7
Water	35.5	31.3
R.m.s. deviations		
Bond lengths (Å)	0.011	0.014
Bond angles (°)	1.587	1.701
Ramachandran plot		
Most favored (%)	97.9	98.3
Additional allowed (%)	1.9	1.7
Outlier (%)	0.2	0

*Values in parentheses are for highest-resolution shell.
